# Psychometric properties of the Arabic version of the Intuitive Eating Scale-2 (IES-2) in a sample of community adults

**DOI:** 10.1186/s40337-023-00782-3

**Published:** 2023-04-01

**Authors:** Feten Fekih-Romdhane, Chen Jiang, Sahar Obeid, Diana Malaeb, Nancy Chammas, Mirna Fawaz, Michel Soufia, Runtang Meng, Souheil Hallit

**Affiliations:** 1 The Tunisian Center of Early Intervention in Psychosis, Department of Psychiatry “Ibn Omrane”, Razi Hospital, 2010 Manouba, Tunisia; 2 Faculty of Medicine of Tunis, Tunis El Manar University, Tunis, Tunisia; 3 School of Public Health, Hangzhou Normal University, Hangzhou, 311121 People’s Republic of China; 4 Social and Education Sciences Department, School of Arts and Sciences, Lebanese American University, Jbeil, Lebanon; 5 College of Pharmacy, Gulf Medical University, Ajman, United Arab Emirates; 6 School of Pharmacy, Lebanese International University, Beirut, Lebanon; 7 School of Medicine and Medical Sciences, Holy Spirit University of Kaslik, P.O. Box 446, Jounieh, Lebanon; 8 Faculty of Health Sciences, Beirut Arab University, Tareek Al Jadida, Afeef Al Tiba, Beirut, 1105 Lebanon; 9 Engineering Research Center of Mobile Health Management System, Ministry of Education, Hangzhou, 311121 People’s Republic of China; 10 Psychology Department, College of Humanities, Effat University, Jeddah 21478, Saudi Arabia; 11 Research Department, Psychiatric Hospital of the Cross, Jal Eddib, Lebanon; 12 Applied Science Research Center, Applied Science Private University, Amman, Jordan

**Keywords:** Intuitive eating, IES-2, Psychometric properties, Arabic, Validation

## Abstract

**Background:**

There is a growing attention on intuitive eating (IE) styles in the Western world that has not yet reached Arab countries, which is likely due to the lack of psychometrically sound measures of the IE construct for Arabic-speaking people. The current study aims to examine the psychometric properties of an Arabic translation of the most widely used measure of IE—the Intuitive Eating Scale-2 (IES-2), in an Arabic-speaking community population from Lebanon.

**Methods:**

Two samples of Arabic-speaking community adults from Lebanon (sample 1: n = 359, 59.9% females, age 22.75 ± 7.04 years; sample 2: n = 444, 72.7% females, age 27.25 ± 9.53 years) were recruited through online convenience sampling. The translation and back-translation method was applied to the IES-2 for linguistic validation. Factorial validity was investigated using an Exploratory Factor Analysis & Confirmatory Factor Analysis strategy. Composite reliability and sex invariance were examined. We also tested convergent and criterion-related validity through correlations with other theoretically plausible constructs.

**Results:**

Nine out of the original 23 items were removed because they either loaded below 0.40 and/or cross-loaded too highly on multiple factors. This resulted in four domains (Unconditional Permission to Eat, Eating for Physical Rather than Emotional Reasons, Reliance on Hunger and Satiety Cues, and Body-Food Choice Congruence) and 14 items retained. Internal reliability estimates were excellent, with McDonald’s ω values ranging from 0.828 to 0.923 for the four factors. Multigroup analysis established configural, thresholds, metric, scalar, strict invariance across gender. Finally, higher IES-2 total scores were significantly correlated with lower body dissatisfaction scores and more positive eating attitudes, thus attesting to convergent and criterion-related validity of the scale.

**Conclusions:**

The current findings provide preliminary evidence for the appropriate psychometric qualities of the Arabic 14-item, four-factor structure IES-2; thereby supporting its use at least among Arabic-speaking community adults.

## Abstract (summary)

Diet restriction has proven costly and ineffective in promoting long-term weight loss. As an alternative, innovative behavioral approaches have been proposed, such as intuitive eating (IE) practices. IE focuses on enhancing intrinsic motivation for eating healthily, thus creating effective, sustainable lifestyle changes. The Intuitive Eating Scale-2 (IES-2) is the most widely used measure to assess IE in patients and the general population. This scale explores four components of IE: *Unconditional Permission to Eat* (i.e., not trying to stave off hunger and refusing to label certain foods as forbidden), *Eating for Physical Rather than Emotional Reasons* (i.e., eating when physically hungry rather than for emotional reasons), *Reliance on Hunger and Satiety Cues* (i.e., trusting one’s own internal satiety and hunger cues and relying on them to guide eating behaviors), and *Body-Food Choice Congruence* (i.e., the extent to which individuals make food choices that promote their body functioning and performance). Since its development, the IES-2 has been translated into different languages and validated in different countries and populations. However, no Arabic version of the IES-2 is yet available. We proposed in the current study to examine the psychometric properties of an Arabic translation of the IES-2 in an Arabic-speaking community population from Lebanon. Findings provided preliminary evidence for the appropriate psychometric qualities of the Arabic IES-2; thereby supporting its use at least among Arabic-speaking community adults.

## Introduction

In today’s societies, it becomes challenging to self-regulate eating behaviors due to the constant availability of large varieties of food, the over-abundance of environmental cues (such as the appetizing food advertisements), as well as the proliferation of unattainable thin beauty ideals and the diet industry. Therefore, eating habits and behaviors have gradually become detached from satiety and hunger cues [1] and guided by thinness pressures and dieting; which may be potentially harmful to both physical and mental health [2–4]. Restricted-energy diets have proven to trigger body composition changes, to be ineffective in decreasing body mass in the long term [5, 6]; and to even contribute to disordered eating over time [7]. While research on this topic remains scarce in Arab countries, some available evidence has shown that globalization and substantial sociocultural changes gave rise to the thin ideal and increased risk for dieting-, and disturbed-eating behaviors [8]. For instance, previous studies emerging from the Arab world documented high prevalence rates of binge eating (e.g., [9–11]), retrained eating (e.g., [12, 13]), and emotional eating (e.g., [14–16]). Previous findings also suggested that around 40% of the Arab adolescent and adult population of both genders is on a diet [8]. Efforts aiming at overcoming these eating and dieting disorders have been recently directed to innovative behavioral approaches focusing on enhancing intrinsic motivation for eating healthily and creating effective, sustainable lifestyle changes, such as intuitive eating (IE) practices [17–19].

IE refers to adaptive eating behaviors that cultivate a positive and healthy relationship between one’s body and food by relying on internal (satiety and hunger) rather than external (situational and emotional) cues [20]. Specifically, IE consists of fostering conscientious avoidance of emotional eating, recognition and response to bodily sensations of hunger/fullness, acceptance for all body sizes and shapes, food choices that are satisfying but healthy, and rejection of labeling food as “bad” or “good” [21]. For all these characteristics, IE has been consistently found to positively correlate with multiple physical (e.g., improved blood pressure and cholesterol levels) and psychological (e.g., lower levels of depression, better body image, acceptance, esteem, increased satisfaction with life and optimism) health indicators [22]. IE has also been demonstrated to contribute to various eating-related outcomes, including more positive eating attitudes [23], weight gain prevention and weight maintenance [22], lower risk of developing eating disorders, emotional eating, and uncontrolled eating [24].

The Intuitive Eating Scale-2 (IES-2; [25]) is the most widely used measure to assess IE; which represents a revision of the original 21-item three-factor structure Intuitive Eating Scale (IES) developed by Tylka in 2006 [26]. The IES-2 was intended to address some limitations of the earlier version, such as evaluating the presence rather than the absence of intuitive eating attitudes and behaviors, and integrating “gentle nutrition” (i.e., the tendency to make food choices that honor bodies’ needs) as an important component of IE in addition to those already included in the IES [25]. As such, the 23-item IES-2 led to a factor structure composed of four dimensions based on exploratory factor analysis (EFA): *Unconditional Permission to Eat* (i.e., not trying to stave off hunger and refusing to label certain foods as forbidden; 6 items), *Eating for Physical Rather than Emotional Reasons* (i.e., eating when physically hungry rather than for emotional reasons; 8 items), *Reliance on Hunger and Satiety Cues* (i.e., trusting one’s own internal satiety and hunger cues and relying on them to guide eating behaviors; 6 items), and *Body-Food Choice Congruence* (i.e., the extent to which individuals make food choices that promote their body functioning and performance; 3 items). The parent study has demonstrated adequate fit indices of this four-dimensional model of IE via confirmatory factor analysis (CFA), as well as good reliability and validity in a sample of male and female university students from the United States [25].

Since its development, many studies supported the psychometric properties of the IES-2 in different countries and languages, including French [27, 28], Italian [29], Greek [30, 31], German [32, 33], Hungarian [34], Polish [35], Portuguese [36, 37], Persian [38], Malay [39], Chinese [40], Romanian [41], and Turkish [42]. The psychometric properties of the IES-2 were also upheld in different age groups (e.g., Adolescents [43, 44], Older adults [45]), in low-income and racial minority populations [46], in culturally diverse samples (e.g., adult Latina women [47], Hispanic American adults [48]), and in a range of clinical groups (e.g., patients with eating disorders [33, 49], breast cancer survivors [38] and “adolescents with overweight/obesity” [44]). In particular, findings from previous validations were consistent to demonstrate good internal reliability of the IES-2 [27, 32, 37, 50]; as well as its construct validity as evidenced through adequate patterns of correlations with relevant constructs (e.g., disordered eating symptoms [28, 32, 33, 36, 50], body image disturbances [25, 28, 33, 36–38], and psychological well-being [27, 28, 32, 50]). Evidence of measurement invariance across sex of the IES-2 was also provided in some samples (adults of Portuguese [36, 37], Malay, Chinese [39], and Romanian [41] origin); thus confirming that IES-2 items are interpreted in the same manner by males and females. However, previous findings on factorial validity of the IES-2 were rather mixed, with some linguistic validation studies having confirmed the 4-factor solution proposed in the original study [28, 32, 33, 36–38, 42, 50], and others failing to support this factorial structure [27, 41, 46, 47].

To date, no Arabic version of the IES-2 is yet available. We could find only one IE measure that has been translated to Arabic and validated in 2004 among Jordanian university students, i.e. the Intuitive Eating Scale by Hawks et al. [51, 52]; which contains 27 items divided into four subscales (intrinsic eating, extrinsic eating, anti-dieting, and self-care). However, this scale had some flaws, with the intrinsic eating subscale having shown both low test–retest reliability and internal consistency, and the self-care subscale having failed to yield desired results regarding the concurrent and construct validity [52]. This might explain its limited use in scientific research. We could find only a very few studies on IE conducted in Arab countries, using either Hawks et al.’ s scale [53, 54] or self-developed measures [55]. This highlights the strong need to validate the largely used IES-2 in the Arabic language, to allow for cross-cultural comparisons, and help validate the current hypothesized four-dimension model of IE in different cultural backgrounds.

In this regard, we propose in the current study to examine the psychometric properties of an Arabic translation of the IES-2 in an Arabic-speaking community population from Lebanon. To that end, we investigated factorial validity using an EFA-to-CFA strategy [56], composite reliability, invariance across sex (i.e., males and females). We also tested convergent and criterion-related validity through correlations with other theoretically plausible constructs. We expected that: (1) the Arabic IES-2 will show good reliability and validity, (2) the four-factor structure will be supported and invariant between genders, and (3) the validity of the scale will be evidenced by appropriate patterns of correlations with BMI, body dissatisfaction, and eating attitudes scores.

## Methods

### Participants

The description of the two samples is summarized in Table 1, with a significant higher number of females and a higher mean age found in sample 2.

**Table 1:** Sociodemographic and other characteristics of the participants

	Sample 1 (n = 359)	Sample 2 (n = 444)	X^2^* / t*	*p*
Gender			14.85	**< .001**
Male	144 (40.1%)	121 (27.3%)		
Female	215 (59.9%)	323 (72.7%)		
Age, in years	22.75 ± 7.04	27.25 ± 9.53	7.68	**< .001**
Body Mass Index, kg/m^2^	24.12 ± 5.13	23.84 ± 4.29	.82	.412

Bold fonts represent significance at *p* < 0.05

### Study design

Data of sample 1 was collected between October and November 2022 (cross-sectional study), whereas that of sample 2 was collected between June and July 2021 (a second cross-sectional study). All participants were recruited conveniently, using a Google Form link. The research team members sent the link to people they know using social media applications (WhatsApp, Facebook, Instagram); those people were then asked to forward the link to other friends and family members they know. Lebanese adult citizens (aged 18 years and above) of both genders (i.e., males and females) and residents of the country were eligible to participate. IP addresses were examined to ensure that no participant took the survey more than once. An introductory paragraph was inserted at the beginning, which explained the purpose of the current study, ensured anonymity of the participant, and the voluntariness of consent to research. After providing digital informed consent, participants were asked to complete the instruments described above, which were presented in a pre-randomised order to control for order effects. The survey was anonymous and participants completed the survey voluntarily and without remuneration.

### Measures 

#### The Intuitive Eating Scale-2 (IES-2)

The Arabic version of the 23-item IES-2 [25] was administered to all participants. The translation procedure of the IES-2 is described above. Items were rated on a 5-point scale, ranging from 1 (strongly disagree) to 5 (strongly agree). Higher mean values refer to higher levels of adaptive, intuitive eating patterns and behaviors.

#### The Eating Attitude Test-7 (EAT-7)

This scale is a shortened version of the Eating Attitude Test-26 [57, 58], which has recently been validated in Arabic by our team [59]. It is a seven-item single-factor structure measure. Each item can be rated on a four-point Likert scale from 0 (infrequently/almost never/never) to 3 (always). Total scores range from 0 to 21, with higher scores indicating greater disordered eating attitudes. Our sample yielded a McDonald’s ω of 0.87.

#### The body dissatisfaction subscale of the Eating Disorder Inventory-second version (EDI-2)

The Eating Disorder Inventory (EDI-2) dimension “body dissatisfaction” [60] was used to evaluate respondents’ dissatisfaction with their body parts and overall body shape. This is a 9-item measure, scored on a 4-point Likert scale, ranging from 0 (sometimes, rarely, never) to 3 (always). Higher total scores reflect higher levels of body dissatisfaction [60]. This measure has previously been used to assess body dissatisfaction in the Lebanese general population (e.g., [61, 62]). In the present sample study, McDonald’s ω was 0.69.

### Translation procedure 

The IES-2 was translated to the Arabic language with the purpose of achieving semantic equivalence between measures in their original and Arabic versions following international norms and recommendations [63]. For this, the forward–backward translation method was applied. The English version was translated to Arabic by a Lebanese translator who was completely unrelated to the study. Afterwards, a Lebanese psychologist with a full working proficiency in English, translated the Arabic version back to English. The translation team ensured that any specific and/or literal translation was balanced. The initial and translated English versions were compared to detect/eliminate any inconsistencies and guarantee the accuracy of the translation by a committee of experts composed of the research team and the two translators [64]. An adaptation of the measure to our specific context was performed, and sought to determine any misunderstanding of the items wording as well as the ease of items interpretation; and, therefore, ensure the conceptual equivalence of the original and Arabic scales in both contexts [65]. After the translation and adaptation of the scale, a pilot study was done on 20 individuals to ensure all questions were well understood; no changes were applied after the pilot study.

### Statistical analysis

We assessed measurement properties following the COnsensus-based Standards for the selection of health Measurement INstruments (COSMIN) guidelines [66–68]. R (version 4.1.2) and its compiler RStudio were used for all data analyses with packages “*MVN*” [69], “*psych*” [70], “*lavaan*” [71], “*semTools*” [72], and “*ufs*” [73, 74]. The data was defined as “exploratory data (*N* = 359)” and “confirmatory data (*N* = 444)” for diverse analyses.

### Structural validity

Following the recommendation of Swami and Barron [56], an examination of the factorial validity structure of the Arabic IES-2 was undertaken using a two-step analytic strategy consisting of exploratory factor analysis (EFA) followed by Confirmatory factor analysis (CFA) on two different samples. Therefore, to shorten items and explore the structure of the IES-2, EFA was applied using exploratory data on sample 1 (*N* = 359) [75]. Prior to EFA, Kaiser–Meyer–Olkin (KMO) test, and Bartlett’s test were performed to check the accessibility for EFA of the data [76, 77]. KMO value higher than or equal to 0.800 and Bartlett’s test is found significant (*p* < 0.050) were favored for conducting EFA. Then, EFA was initially conducted on a full item pool with Promax rotations using the maximum likelihood factoring method [78]. The item would be considered for removal if any of the following criteria were met: 1) the target-loading is less than 0.450; 2) the cross-loading is higher than 0.320 or the gap between target-loading and possible cross-loading is greater than 0.200 [77, 79]. After removing unsatisfactory item(s), EFA would be consequently conducted on the remaining item pool to explore the item belongings of revised scale and provide the possible structural model of the revised IES-2.

CFA was then applied to validate factorial model and identify the relative better factor structure in confirmatory data on sample 2 (*N* = 444) [80, 81]. The weighted least square mean and variance adjusted (WLSMV) estimator was used to adapt the ordinal properties of the data [82–84]. Goodness-of-fit (GOF) indices are considered acceptable if Comparative Fit Index (CFI) higher than 0.900, Tucker-Lewis Index (TLI) higher than 0.900, and Root Mean Square Error of Approximation (RMSEA) lower than 0.080 [85]. The final confirmed factor model would be used in the subsequent analytic approaches.

### Sex measurement invariance

Configural, threshold, metric, scalar, and strict invariance model is built on sex to analyze sex measurement invariance [72, 83]. All parameters of the configural invariance model were set free. Threshold invariance constrained the threshold parameter to test equality. Threshold and factor loadings were constrained to test whether they remain equal in metric invariance model. Besides threshold and factor loadings, scalar invariance further added restrictions on intercept parameter. Last, the strict invariance model restricts the threshold, factor loadings, intercepts, and residuals to determine equivalence. Measurement invariance could be considered supportive in the model if the following GOF indices [86] and their changes within (Δ) were met: CFI > 0.900, TLI > 0.900, and RMSEA < 0.080; ΔCFI ≤ 0.010, ΔTLI ≤ 0.010, ΔRMSEA ≤ 0.015 [87–89].

### Internal consistency

Composite reliability in both subsamples was assessed using McDonald’s ω and its associated 95% CI, with values greater than 0.70 reflecting adequate composite reliability [90]. To assess convergent and criterion-related validity, we examined bivariate correlations between IES-2 scores and those on the additional measures included in the survey (functionality appreciation, body appreciation, disordered eating attitudes, and orthorexia nervosa) using the total sample. All scores had normal distribution, as identified by skewness and kurtosis values varying between − 1.96 and + 1.96 [91]; therefore, Pearson correlation test was used. Based on Cohen’s recommendations [92], values ≤ 0.10 were considered weak, ~ 0.30 were considered moderate, and ~ 0.50 were considered strong correlations.

## Results

### Structural validity

Initial EFA with promax rotation on the original item set of IES-2 (KMO = 0.903; Bartlett’s test *χ*^*2*^ (253) = 4585.543, *p* < 0.001) yielded a five-factor structure with 57.819% of the total variance (Table 2). The loading was found below 0.450 in item 09 (“I use food to help me soothe my negative emotions”), item 13 (“When I am lonely, I do NOT turn to food for comfort”), item 14 (“I find other ways to cope with stress and anxiety than by eating”), item 18 (“I rely on my hunger signals to tell me when to eat”), and item 21 (“Most of the time, I desire to eat nutritious foods”). Moreover, the cross-loading of item 04 (“I use food to help me soothe my negative emotions”) was found higher than 0.320 and possible cross-loading of item 12 (“When I am lonely, I do NOT turn to food for comfort”) and item 22 (“I find other ways to cope with stress and anxiety than by eating”) were noticed regarding they are close to the cut-off (-0.270 and 0.275, respectively). Given gaps between target loading and possible cross-loading of items 12 and 22 are both higher than 0.200 (0.344 and 0.277, respectively), the two items were still chosen for removal. In sum, accounted nine items in total, item 04, 09, 12, 13, 14, 18, 21, 22, and 23 were removed from the IES-2–23 due to low factor loadings or high cross-loading.

**Table 2:** Factor loadings for each item in the exploratory factor analysis of exploratory data (*N* = 359)

Variables	Factor 4	Factor 1	Factor 2	Factor 5	Factor 3	Communalities	Uniquenesses	Complexity
IES-2 01	**− 0.302**	0.020	0.100	**0.712**	0.126	0.450	0.550	1.467
IES-2 02	0.053	− 0.017	**0.836**	0.131	− 0.066	0.700	0.300	1.071
IES-2 03	**0.825**	− 0.130	0.170	0.145	0.025	0.519	0.481	1.204
IES-2 04 ^a^	− 0.131	− 0.175	**0.336**	**0.621**	− 0.037	0.392	0.608	1.842
IES-2 05	0.137	− 0.065	**0.667**	− 0.005	0.007	0.465	0.535	1.104
IES-2 06	0.038	**0.737**	− 0.071	0.041	0.019	0.638	0.362	1.032
IES-2 07	− 0.141	**0.917**	0.074	− 0.007	0.064	0.728	0.272	1.070
IES-2 08	− 0.052	**0.957**	− 0.004	− 0.018	− 0.091	0.749	0.251	1.025
IES-2 09^a^	0.150	0.214	0.146	**0.349**	− 0.233	0.236	0.764	3.391
IES-2 10	− 0.038	0.094	**0.829**	0.118	− 0.012	0.697	0.303	1.071
IES-2 11	0.000	0.012	**0.783**	0.041	0.060	0.634	0.366	1.018
IES-2 12^a^	**0.551**	0.048	*− 0.270*	0.207	0.012	0.582	0.418	1.790
IES-2 13^a^	**0.377**	0.039	− 0.140	**0.319**	0.064	0.467	0.533	2.339
IES-2 14^a^	**0.393**	0.069	− 0.070	**0.380**	− 0.077	0.453	0.547	2.207
IES-2 15	**0.752**	− 0.124	− 0.208	0.244	− 0.041	0.653	0.347	1.444
IES-2 16	**0.966**	− 0.057	0.107	− 0.272	0.008	0.708	0.292	1.191
IES-2 17	**0.711**	− 0.015	0.124	− 0.146	0.007	0.439	0.561	1.149
IES-2 18^a^	0.274	− 0.034	0.046	0.253	**0.364**	0.525	0.475	2.769
IES-2 19	− 0.017	0.098	0.009	0.163	**0.725**	0.747	0.253	1.141
IES-2 20	0.142	− 0.046	− 0.034	0.023	**0.855**	0.863	0.137	1.066
IES-2 21^a^	**0.307**	0.295	0.081	− 0.045	0.203	0.484	0.516	2.933
IES-2 22^a^	**0.552**	*0.275*	0.096	− 0.088	− 0.014	0.545	0.455	1.600
IES-2 23^a^	**0.316**	**0.566**	0.030	− 0.126	0.020	0.624	0.376	1.695
SS loadings	4.155	2.991	2.807	1.592	1.753	NA	NA	NA
Proportion Var	0.181	0.130	0.122	0.069	0.076	NA	NA	NA
Cumulative Var	0.181	0.311	0.433	0.502	0.578	NA	NA	NA
Proportion explained	0.313	0.225	0.211	0.120	0.132	NA	NA	NA
Cumulative proportion	0.313	0.537	0.748	0.868	1.000	NA	NA	NA

Bold font indicates items loadings higher than 0.300. Italic font indicates the possible cross-loadings of items on the factor

^a^Items to be removed

A four-factor structure was then found with the follow-up EFA (KMO = 0.848; Bartlett’s test *χ*^*2*^ (91) = 2564.593, *p* < 0.001) on the remaining 14 items (Table 3), accounting 61.774% of the total variance. As no factor-loading of items was found below 0.450 and no cross-loading of items was detected, the possible form of the revised IES-2 was confirmed as a four-factor structure: factor one including item 02, 05, 10, and 11; factor two including item 03, 15, 16, and 17; factor three including item 06, 07, and 08; and factor four including item 01, 19, and 20.

**Table 3:** Factor loadings for each item in the exploratory factor analysis of exploratory data (*N* = 359)

Variables	Factor 2	Factor 4	Factor 1	Factor 3	Communalities	Uniquenesses	Complexity
IES-2 01	0.102	− 0.237	0.012	**0.471**	0.180	0.821	1.587
IES-2 02	**0.830**	0.021	− 0.012	− 0.016	0.691	0.309	1.002
IES-2 03	0.091	**0.751**	− 0.040	0.016	0.576	0.424	1.036
IES-2 05	**0.646**	0.123	− 0.058	− 0.006	0.454	0.546	1.089
IES-2 06	− 0.066	0.085	**0.698**	0.069	0.632	0.369	1.068
IES-2 07	0.097	− 0.048	**0.860**	0.046	0.749	0.252	1.038
IES-2 08	− 0.002	0.040	**0.897**	− 0.092	0.758	0.242	1.025
IES-2 10	**0.851**	− 0.032	0.093	0.000	0.721	0.279	1.027
IES-2 11	**0.789**	0.006	− 0.012	0.048	0.638	0.362	1.008
IES-2 15	− 0.239	**0.483**	0.038	0.256	0.460	0.540	2.058
IES-2 16	0.014	**0.892**	0.013	− 0.042	0.774	0.226	1.005
IES-2 17	0.065	**0.591**	0.051	0.029	0.427	0.573	1.044
IES-2 19	0.023	0.017	0.028	**0.865**	0.800	0.200	1.004
IES-2 20	− 0.012	0.174	− 0.053	**0.818**	0.789	0.212	1.100
SS loadings	2.558	2.185	2.091	1.814	NA	NA	NA
Proportion Var	0.183	0.156	0.149	0.130	NA	NA	NA
Cumulative Var	0.183	0.339	0.488	0.618	NA	NA	NA
Proportion explained	0.296	0.253	0.242	0.210	NA	NA	NA
Cumulative proportion	0.296	0.549	0.790	1.000	NA	NA	NA

Bold font indicates items loadings higher than 0.300

*NA* not applicable

**Fig. 1:**
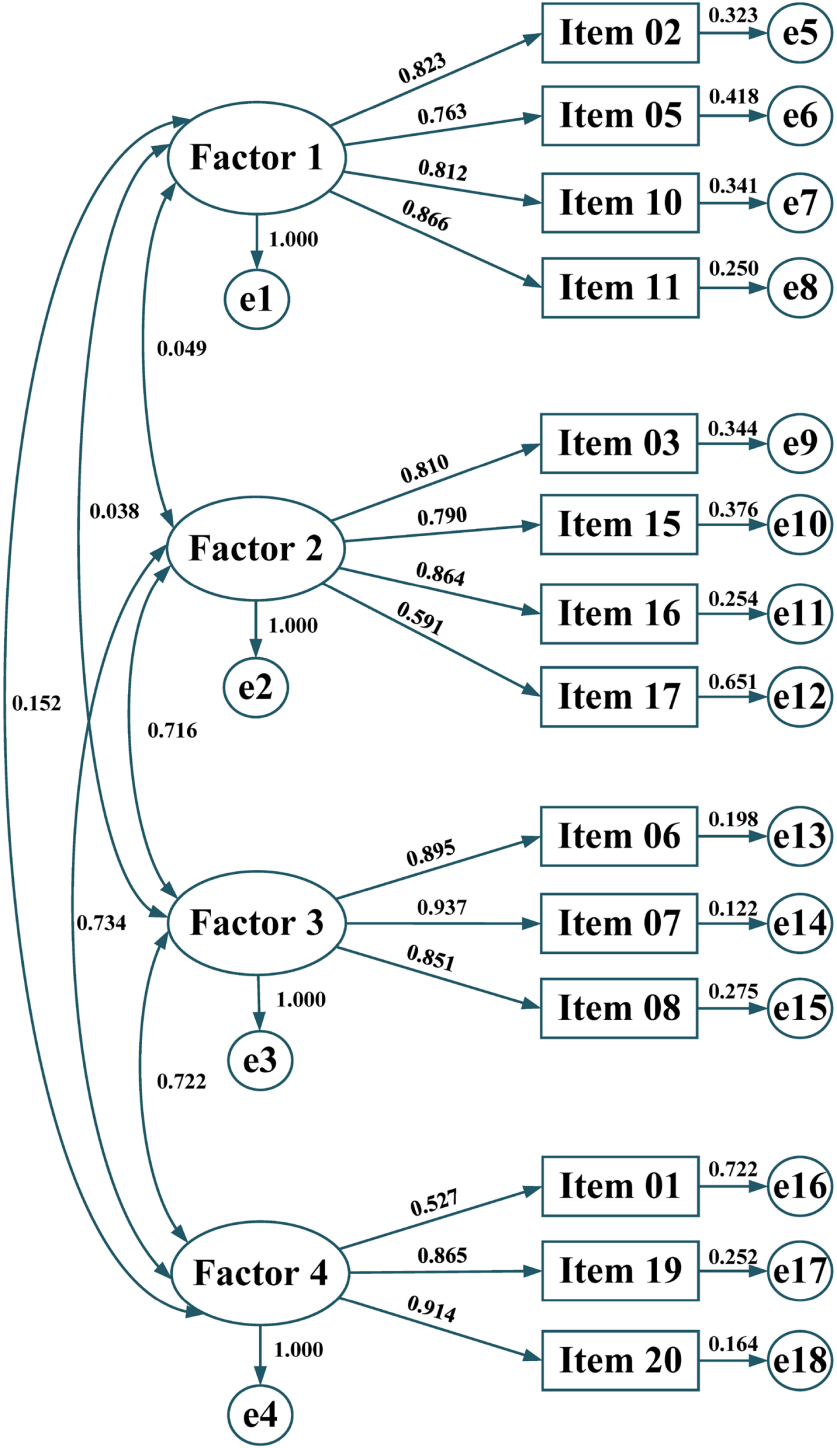
Standardized coefficients of confirmatory factor analysis results for a four-factor model of the IES-2. *Note*: The one-sided edges represent coefficient values (i.e., factor loadings) while the double-sided one the covariance between two factors

On the basis of the EFA analysis, CFAs were then conducted to examine three possible models of the IES (Table 4). Compared to the one-factor and second-order factor models, GOF indices of the four-factor model showed a better fit with a CFI of 0.945, a TLI of 0.929, and an RMSEA of 0.130. Therefore, the final four-factor (Eating for Physical Rather than Emotional Reasons [F1], Unconditional Permission to Eat [F2], Reliance on Hunger and Satiety Cues [F3], and Body-Food Choice Congruence [F4]) structure of the revised IES-2 was confirmed and formed for further analysis (Fig. 1).

**Table 4:** Confirmatory factor analysis outcomes of the revised IES-2 (*N* = 444)

Model	IES-2				
	*χ*^*2*^	*df*	CFI	TLI	RMSEA (90% CI)
One-factor model	3466.604	77	0.648	0.584	0.315 (0.306, 0.324)
**Four-factor model**	**603.284**	**71**	**0.945**	**0.929**	**0.130 (0.121, 0.140)**
Second-order factor model	5879.780	77	0.397	0.288	0.412 (0.404, 0.421)
Threshold			> 0.900	> 0.900	< 0.080

Bold font stands for the best fit model

*IES*, Intuitive Eating Questionnaire; *χ*^*2*^, Chi-square; *df*, degrees of freedom; CFI, Comparative Fit Index; TLI, Tucker-Lewis Index; RMSEA, root mean square error of approximation; CI, confidence interval

### Sex measurement invariance

Sex measurement invariance was therefore analyzed with the confirmed IES-2. Most GOF indices of sex measurement invariance kept within an acceptable range, except RMSEA values slightly fell outside the cutoff criteria. As all the ΔCFI, ΔTLI, and ΔRMSEA were below cut-off values, all sex measurement invariance models were determined to be fully supported (Table 5). No significant difference was found between males and females in terms of IES scores (42.83 ± 3.60 vs. 42.30 ± 3.59; t (442) = 1.367; *p* = 0.172; Table 1).

**Table 5:** Sex measurement invariances of the revised IES-2 (*N* = 444)

Hypothesis	*χ*^*2*^ (*df*)	Δ*χ*^*2*^ (Δ*df*)	CFI	ΔCFI	TLI	ΔTLI	RMSEA (90% CI)	ΔRMSEA
Sex (male vs. female)								
Configural model	620.993 (142)***		0.950		0.936		0.124 (0.114, 0.134)	
Thresholds model	666.415 (170)***	36.660 (28)	0.949	− 0.001	0.945	0.009	0.115 (0.106, 0.124)	− 0.009
Metric model	676.862 (180)***	19.173 (10)*	0.949	0.000	0.948	0.003	0.112 (0.103, 0.121)	− 0.003
Scalar model	676.026 (190)***	12.113 (10)	0.950	0.001	0.952	0.004	0.108 (0.099, 0.116)	− 0.004
Strict model	697.224 (204)***	37.581 (14)**	0.949	− 0.001	0.954	0.002	0.105 (0.096, 0.113)	0.000
Threshold			> 0.900	< 0.010	> 0.900	< 0.010	< 0.080	< 0.015

*χ*^*2*^, Chi-square; *df,* degrees of freedom; CFI, Comparative Fit Index; TLI, Tucker-Lewis Index; RMSEA, Root Mean Square Error of Approximation; Δ, a change in *χ*^*2*^, *df,* CFI, TLI, and RMSEA

***p < 0.001; **p < 0.010, *p < 0.050

### Internal consistency

The detailed internal consistency information of the revised IES-2 is listed in Table 6. As indicated by McDonald’s ω, the internal consistency of the IES-2 was good all values were higher than the acceptable cut-off value with a range of 0.828‒0.923.

**Table 6:** Internal consistency of the revised IES-2 (*N* = 444)

Variable	McDonald’s ω (95% CI)
IES	0.860 (0.841, 0.879)
F1	0.888 (0.871, 0.905)
F2	0.850 (0.828, 0.873)
F3	0.923 (0.911, 0.935)
F4	0.828 (0.801, 0.855)

This table shows ordinal versions of McDonald’s ω

*IES-2* Intuitive Eating Scale

### Convergent and criterion-related validity

Higher intuitive eating total scores were significantly correlated with lower body dissatisfaction scores and lower EAT-7 scores (more appropriate eating), but not age or BMI (Table 7).

**Table 7:** Bivariate correlations between Intuitive Eating Scale and subscales scores and other measures included in the study and age

	1	2	3	4	5	6	7	8
1. IES total	1							
2. IES Factor 1	0.10*	1						
3. IES Factor 2	0.32***	0.03	1					
4. IES Factor 3	0.66***	− 0.34***	− 0.05	1				
5. IES Factor 4	0.77***	− 0.23***	− 0.08	0.47***	1			
6. Body dissatisfaction	− 0.18***	0.24***	0.10*	− 0.25***	− 0.29***	1		
7. Eating attitudes (EAT-7)	− 0.30***	0.23***	0.10*	− 0.27***	− 0.44***	0.59***	1	
8. Age	− 0.06	0.04	0.03	− 0.06	− 0.08	0.14**	0.19***	1
9. Body Mass Index	− 0.03	0.11*	0.05	− 0.10*	− 0.06	0.09	0.13**	0.31***

*IES*Intuitive Eating Scale, *EAT-7* Eating Attitudes Test-7

*p < .05; **p < .01; ***p < .001

## Discussion

The purpose of the present research was to translate, adapt and validate the IES-2 into the Arabic language in an adult sample derived from the Lebanese general population. Our results showed that the Arabic version of the scale reflects the original factorial structure, albeit with lesser number of items. Internal reliability estimates of all IES-2 factors were excellent. Evidence for convergent and Criterion-Related Validity has also been supported although the correlations were in the low-to-medium range. Findings suggest the Arabic IES-2 to be a valid and reliable measure for the evaluation of IE patterns. Accordingly, we recommend its use in Arab contexts.

In the current study, CFA and EFA were performed using two different sample data as recommended in the literature [93], thus enabling an independent cross-validation of the factor structure of the Arabic IES-2. Items that loaded below 0.40 were considered to insufficiently represent a factor and were therefore removed [94]. Items that cross-loaded too highly on multiple factors were also dropped [95]. This has led to a removal of nine out of the original 23 items, resulting in four domains and 14 items retained. All remaining items had satisfactory factor loadings. Previous validation studies showed that there is some ambiguity surrounding the factorial validity of the IES-2. While some studies corroborated, through CFA, the four-dimension model proposed by the developers of the scale (e.g., [28, 32, 33, 36, 38, 42]), others failed to confirm this original factorial structure and rather retained a three- [27, 41], five- [47] or six- [46] factor model. In agreement with our findings, previous studies with Turkish [50] and Brazilian [37] adults were able to replicate the parent 4-factor model of IES-2 scores only after omission of several items. In the Turkish version, items 6 and 21 were removed [50], while in the Brazilian version, items 1, 10, 13, and 15 were excluded [37]. These discrepancies may be explained by inter-ethnic and trans-cultural differences in the construct of IE [41, 50, 96]. Indeed, people from Arab backgrounds may have different eating habits and patterns that are different than other cultural groups. For example, eating patterns and disorders are often expressed by, and attributed to, somatic complaints in Arab as compared to Western people [97, 98]. Individuals in Arab cultures tend also to be highly connected emotionally and culturally to food [99]; as such, they spend a large amount of their incomes on their food [100]. The lifestyle in Arab cultures is closely attached to food, with most of the social and religious celebrations being accompanied by the consumption of large amounts of food [41]. In addition, the vast majority of Arab people are Muslims, a religion that supports restrictive eating [101]. All these peculiarities might have affected the factorial validity of the proposed model of the IES-2 in our sample. In sum, the factorial structure of IES-2 scores is still under debate and future cross-cultural research is needed to confirm its robustness across diverse national and social groups.

The Arabic IES-2 presented good internal consistency, with McDonald’s ω values ranging from 0.828 to 0.923 for the four factors. McDonald’s ω was selected as a measure of composite reliability because of known problems with the use of Cronbach’s α [102]. In the original validation [25], as well as most of the previous studies on other linguistic validations of the scale, internal reliability was examined using Cronbach alpha estimates and showed adequate values (e.g., Turkish: α = 0.71–0.94 [50]; Portuguese: α = 0.79–0.89 [37]; French: α = 0.70–0.92 [27]; Hungarian: α = 0.84–0.89; German: α = 0.87–0.95 [32]). Furthermore, multigroup analysis established configural, metric, and scalar invariance across gender. So far, a very few studies have specifically investigated sex invariance of IES-2 scores. The Portuguese versions showed only metric sex invariance [37] or full scalar invariance [36] in community adults. In line with our findings, the Malay [39] and the Romanian [41] versions of the IES-2 demonstrated configural, metric, and scalar invariance between sex groups. Providing evidence for measurement invariance of the Arabic IES-2 with respect to sex will allow future clinicians and researchers to use this measure confidently irrespective of respondents’ sex, and enable psychometrically sound comparisons of means between males and females [88, 103]. Our between-groups comparisons revealed no significant sex difference in terms of IES scores. Studies that have explored sex differences have generally found that women exhibited lower IES-2 scores than men, with small-to-medium effect sizes [25]. In contrast, Swami et al. [39] found no significant sex differences in two IES-2 dimensions (Reliance on Hunger and Satiety Cues and Body-Food Choice Congruence); and greater scores among Malay women on the Eating for Physical Rather Than Emotional Reasons dimension, though the magnitude of the difference was small and not meaningful. Furthermore, were found no significant correlations between age and the different facets of IE, which is consistent with previous research (e.g., [33]). We highlight, however, that the vast majority of previous validation studies of the IES-2 did not perform analysis with respect to age.

Finally, higher IES-2 total scores were significantly correlated with lower body dissatisfaction scores and more positive eating attitudes (correlations within low-to-medium range), thus attesting for convergent and Criterion-Related Validity of the scale. The construct validity of IES-2 scores has consistently been supported in the available research through significant negative correlations with disordered eating [28, 32, 33, 36, 50], disturbance in eating attitudes [25, 38], body shame [25], negative body image [28, 33], and significant positive correlations with positive body image [28, 36, 37], body appreciation [38], and psychological well-being [27, 28, 32, 50]. Although, contrary to our findings, previous validation studies demonstrated significant negative correlations between IES-2 scores and BMI (e.g., [36–38]), there is evidence that IE contributes to weight maintenance rather than weight loss [22]; which could explain our findings.

## Limitations and directions for future research

Our findings should be interpreted while bearing in mind the following limitations. The first limitations lie to the cross-sectional design and recruitment method (online convenient sampling of non-clinical adults from Lebanon); which prevent causal inferences and generalization of our findings to the wider Arabic-speaking population. This is especially important given that large differences in food intake habits and eating problems have been documented across Arab countries [104]. Future replication of our results in large Arabic-speaking samples with a cross-national examination of the factorial structure may help address this issue and support the psychometric properties of the Arabic IES-2. Additionally, future validations of the Arabic IES-2 in clinical populations are still required. Another limitation consists of the fact that we did not assess other relevant psychometric properties of the IES-2, such as test–retest reliability and predictive validity. Additional studies should consider addressing this limitation. Finally, similar to some previous IES-2 validations (e.g., German version, 82.6% females [32]; Hungarian version, 80.2% females [34]; Portuguese version, 72.8% females [36]), there was a larger proportion of females (72.7%) in our second sample. This disproportion with respect to sex may be due to the females’ receptivity in completing online surveys. This aspect makes it difficult to generalize our findings. Therefore, there is the need for future research on samples with substantially homogeneous sex distribution in order to have a more accurate validation of the Arabic IES-2, including robust results with respect to the measurement invariance.

## Conclusion

The growing attention on intuitive eating style in the Western world has not yet reached Arab countries, which is likely due to the lack of psychometrically sound measures of the IE construct for Arabic-speaking people. The current findings provide preliminary evidence for the appropriated psychometric qualities of the Arabic 14-item, four-factor structure IES-2; thereby supporting its use among Arabic-speaking adults. Pending future cross-national validation studies in larger clinical and non-clinical samples, making available an Arabic valid version of the IES-2 will hopefully facilitate improved research and clinical practices related to IE in Arabic-speaking nations and communities.

## Data Availability

The authors do not have the right to share any data information as per their institutions policies (copyright issues). Data can be shared upon a reasonable request from the corresponding author (S.H.).
